# Animal Welfare Underenforcement as a Rule of Law Problem

**DOI:** 10.3390/ani12111411

**Published:** 2022-05-30

**Authors:** M. B. Rodriguez Ferrere

**Affiliations:** Faculty of Law, University of Otago, Dunedin 9054, New Zealand; marcelo.rodriguez.ferrere@otago.ac.nz

**Keywords:** law, animal welfare, rule of law, enforcement

## Abstract

**Simple Summary:**

Animal welfare legislation is routinely underenforced by the state. This underenforcement has obvious and direct implications for the animal victims of cruelty and neglect. This article proposes that there are also less obvious constitutional implications of such underenforcement: it undermines the rule of law. This article constructs this argument from a review of rule of law literature and investigates the implications of underenforcement being a constitutional problem.

**Abstract:**

Many have decried the state’s underenforcement of animal welfare legislation because of the direct negative effects on animal interests. This article will advance the argument that such underenforcement has a much deeper societal effect because it undermines the rule of law. It does so by first, reviewing rule of law literature to advance the proposition that the state has a general obligation to enforce the law and, specifically, animal welfare legislation. It then looks to the practical issues that arise with the argument, specifically prosecutorial discretion and private prosecutions. Finally, it concludes that the state’s underenforcement of animal welfare legislation does indeed run contrary to the rule of law, and thus regardless of whether we have the interests of animals at the front of our minds, it is a matter that should concern us all.

## 1. Introduction

The “enforcement gap” between animal welfare legislation as declared, and the actual enforcement of that legislation, is a well-documented phenomenon [1]. It is this gap that allows myriad cases of animal cruelty and failures to provide for an animal’s welfare to go undetected and unprosecuted. The enforcement gap obviously raises the ire of those who are concerned by the gap’s negative effect on lives of animals. This paper intends to assess the gap from a different angle. It will investigate whether it is possible to frame the enforcement gap—the systemic failure by the state to effectively enforce animal welfare legislation—as a constitutional issue, and specifically, a rule of law problem. If the enforcement gap undermines the rule of law, then it is a problem that should concern us all, not simply those who care about the interests of animals, and thus such a framing might provoke greater urgency to close the gap.

## 2. Method, Assumptions and Definitions

In this paper, I have conducted a review of rule of law literature to advance the argument that the underenforcement of animal welfare legislation is unconstitutional. The argument has three sections. First, I want to advance the proposition that one conception of the rule of law demands that, in general, the state has an obligation to enforce the law as declared. Second, I want to advance the proposition that, in particular, the state has an obligation to enforce animal welfare legislation. Finally, I want to address the practical concerns with taking these propositions to their logical conclusion. The analysis focuses on “common law” countries—i.e., those that follow the English tradition, but is of applicability to all countries with animal welfare legislative frameworks.

The key assumption in this paper is that state—directly or indirectly—routinely underenforces animal welfare legislation. It is a safe assumption to make. While there is not space in this paper to go into extensive detail, commentators have already outlined underenforcement of animal welfare legislation in various jurisdictions, including the United States [2,3], Canada [4], and Australia [1]. Empirical data in the form of prosecution statistics is difficult to come by, but in New Zealand, the humane society largely responsible for enforcing its Animal Welfare legislation only prosecutes between 0.27 and 0.4 per cent of the complaints it receives [5,6]. The experience in these jurisdictions is likely replicated in all jurisdictions with animal welfare legislation. The state in these jurisdictions causes this enforcement gap in two main ways: First, to the extent that it uses existing law enforcement infrastructure, by failing to directly enforce the law sufficiently. Second, as shown by the New Zealand example above, divesting the responsibility to enforce the law to non-state actors, in most instances, humane societies [7].

When I use the term ‘enforcement’, I refer to all stages of the enforcement process, from detection to the successful prosecution of offending. This paper is not an argument for what has been termed “progressive carceralism”, and it does not advocate for harsher punishment for animal welfare offending [8]. Enforcement does not necessarily need to involve carceral outcomes, and indeed, sometimes “in the field” educational or supportive responses to offending—as an alternative to a criminal or regulatory response—are more appropriate [9]. However, such responses require the detection of offending in the first place, and a lack of detection is a key part of the enforcement gap. There are obvious policy problems with the underenforcement of animal welfare legislation: as discussed above, it has a direct negative effect on the animals it fails to protect, but more broadly, it undermines the deterrence value that enforcement provides, and therefore undermines the value of the legislation itself. My aim in this paper is to elevate the concern with such underenforcement beyond the political level to the constitutional level. Put simply, I wish to argue that the underenforcement of animal protection/welfare legislation is a constitutional problem.

The constitutional vehicle I wish to use for this elevation is the rule of law. Unfortunately, compared to ‘enforcement’, the ‘rule of law’ is a much harder term to define, and I cannot provide a comprehensive account here: it is an oft-used phrase, to the point of cliché [10]. Whereas once it was easily articulated as one of the “twin pillars” of the British constitution—one that controlled the dangers of unlimited discretionary power [11], it is now invoked so often and in such a range of circumstances that it is “the closest thing we have to a universal political ideal” [12], or, cynically, meaningless. For the purposes of this article, I use the term to help assess “the character of official legal institutions, rules and practices, features taken to be necessary for a legal order to be in good shape” [13]. The problem is that this assessment will often require the use of metrics, and identifying those metrics risks a “laundry list” of attributes, which, if satisfied, indicate adherence to the rule of law [14]. I do not wish to wade into the debate as to which metrics appropriately belong in the laundry list, let alone the meta-debate about how to categorise the positions of those in the debate [15]. For the purposes of this paper, I simply want to use general versions of the two major conceptions: a ‘thinner’ minimalist/formal conception, and a ‘thicker’ maximalist/substantive conception [13] (p. 111).

To show that the enforcement gap detailed above amounts to a rule of law problem, I first need to show that adherence to the rule of law demands enforcement of the law. In the next two sections, I will outline how the state is under a general obligation to enforce the law (using a “thin” conception of the rule of law) and a specific obligation to enforce animal welfare legislation in particular (using a “thick” conception of the rule of law).

## 3. Is the State under a General Obligation to Enforce the Law?

This section will advance the proposition that the state is under a general obligation to enforce the law, using a “thin conception of the rule of law”, which puts a premium on legal certainty and predictability [16]. A legal system that adheres to such a conception might ensure the publicity, stability, consistency and prospectivity of the law, whilst also ensuring congruence between the law on the books and the way in which public order is actually administered [17] (p. 39). Such a conception does not prescribe any substantive requirements, or any particular content of the law. Instead it focuses on ensuring that the law tells a citizen “what facts he may count on and thereby extends the range within which he can predict the consequences of his actions” [18]. There are two aspects in this conception of the rule of law that indicate the state is under an obligation to enforce the law: publicity and congruence.

### 3.1. Publicity of the Law

The first idea is that law needs to be public, which might not seem like an obvious requirement of the rule of law. As Lon Fuller notes:

“Why all this fuss about publishing [laws]? Without reading the criminal code, the citizen knows he shouldn’t murder and steal”.

[17] (p. 51)

One of Fuller’s responses is about the power of the law to shape public behaviour:

“[i]n many activities men observe the law, not because they know it directly, but because they follow the pattern set by others whom they know to be better informed than themselves. In this way knowledge of the law by a few often influences indirectly the actions of many”.

[17] (p. 51)

Similarly, F. A. Hayek argues that the “collaboration of individuals under common rules rests on a sort of division of knowledge” [18] (p. 225). While no one person will know all the law in force at any given moment, collective knowledge about the law will be total, and alter the way individuals within society act. The importance of the publicity of law is further strengthened by Jeremy Waldron’s observations on public norms:

“A system of political rule is not a system of law unless social order is organized around the existence of identifiable norms issued for the guidance of conduct.”

[16] (p. 24)

The state need not disseminate these norms and rules to each individual citizen, simply make them available and accessible [16] (p. 24). When we talk about “the public character of law” we thus talk about “the abiding presence of certain norms in a given society [16] (p. 24).

However, as Waldron continues, the importance of these norms—and law’s capacity to shape them—is not simply “pragmatic administrative convenience along the lines of its being easier to govern people if they know what is expected of them” [16] (p. 24). Instead, it recognises the responsible agency of individuals, and their capacity to modulate their behaviour [16] (p. 25). This is rule by law, rather than rule by terror: the voluntary, self-application of norms and rules by individuals [16] (p. 25). It is crucial for the law to recognise a citizen’s capacity for self-determination, and it does so by being public and knowable, guiding behaviour through the norms that publicity creates.

Given this phenomenon of voluntary self-application, enforcement of the law hardly seems a necessary component of this idea. Indeed, Waldron refers to examples of the vast majority of unsuccessful defendants paying damages without bailiffs seizing their property, and convicts reporting to prison of their own volition [16] (p. 27). Moreover, work on the link between law and social norms has posited that simply publicising law—without any enforcement—will suffice to alter human behaviour [19]. While the state may not enforce the law, “second-order enforcement”, viz. other individuals—neighbours—will enforce the law and the norms it represents by shaming transgressors [20]. 

There are general limitations in such social norm theory [21], but even assuming it is correct, second-order enforcement cannot mean first-order enforcement—i.e., by the state—is irrelevant to the effectiveness of the law. This is demonstrated when other social norms run contrary to the enforcement of the law as stated. In their study of the link between social norms and enforcement, Acemoglu and Jackson note that “laws often go unenforced because they conflict with prevailing social norms”—for example, the law might be archaic, and no longer has any relevance to modern society, even if it is still technically in force [22]. Likewise, enforcement of modern law has the capacity to change archaic norms. They give the example of the United States South in the 1950s, where prevailing racist norms were successfully changed (although not eliminated), not simply through the enactment of antidiscrimination and antiracist law, but through the enforcement of that law [22] (p. 246). Their key conclusion is that

“laws that are in too strong a conflict with the prevailing norms may backfire and significantly increase lawbreaking, whereas more moderate laws that are not in discord with prevailing norms may reduce behavior without causing as much lawlessness—because they change social norms in the process”.

[22] (p. 267)

Unenforced law has the capacity to be effective so long as it aligns with existing social norms, but to change those social norms, it must be enforced. If it is not enforced, and is not aligned with existing social norms, then there is no reason to suppose it will alter human behaviour. Tying this idea to the publicity of the law, if it is critical to ensure that individuals can alter their behaviour accordingly, then the law must have the capacity to alter their behaviour, and that comes through enforcement. From this perspective, in order to uphold the rule of law, the state has an obligation to enforce the law: it must represent a meaningful signal that individuals can rely upon and that social norms reflect. In the absence of enforcement, social norms can run discordant with the law, making it more likely that individuals do not obey the law, and thus making it difficult for other individuals to know what the law is and how order their lives accordingly.

### 3.2. Congruence of the Law

The alternate entry point for enforcement I want to advance is similar to publicity of the law, but focuses on Fuller’s principle of congruence between the law as it is enacted and as it is enforced. This principle is described as one of “commitment”: “by enacting laws a government says to the citizen, “These are the rules we ask you to follow. If you obey them, you have our promise that they are the rules we will apply to your conduct” [17] (pp. 216–217). As Colleen Murphy notes, government failure in their commitment to the declared law by not enforcing it, builds “resentment”, since citizens cannot form reliable expectations due to frequent divergence between the law as declared and its enforcement [23].

“Failures of congruence undermine the confidence with which citizens can look to the written law to determine what officials expect of them. Resentment builds when officials expect citizens to fulfill certain duties, like obedience to law, despite the failure of government officials to fulfill their reciprocal duties”, including the law’s enforcement.

[23] (p. 242)

The product of that resentment is that citizens will have little incentive to comply with the law themselves. In this way, a lack of enforcement of the law by government demonstrates a lack of commitment to the law, and thus undermines its effect. 

Fuller has argued that the idea of congruence between the law as declared and as it is enforced may be the most crucial of conceptions of the rule of law [17]. Others have gone further and argue that it “may seem perverse to focus on anything other than the congruence of enforcement and administration with the law” since, unlike other conceptions, it focuses on the administration of the law, rather than its content or creation [24].

The common denominator between these two aspects—publicity and congruence—is that to give it meaning, the state must be under a general obligation to appropriately enforce the law. If the state fails to enforce the law, then the law’s publicity is meaningless: it will not have the capacity to shape public norms. Similarly, if the state fails to enforce the law, the citizen is under no incentive to comply with it. Either way, the law fails to have effect, and this undermines the rule of law. Systemically underenforced law thus undermines the rule of law.

## 4. Is the State Is under an Obligation to Enforce Animal Welfare Law in Particular?

In Section 3 above, I established that a thin, formalist conception of the rule of law has the capacity to oblige state enforcement of the law. In this section, I want to build on that by advancing a slightly thicker conception that obliges state enforcement of animal welfare legislation in particular.

Thick conceptions of the rule of law distinguish themselves by demanding certain content in the law, for example that the law must “be consistent with the substantive principles of international human rights and fairness as well as the principle that those ruled by the law should also participate in its promulgation” [15] (p. 794). Or, alternatively, the law should recognise “moral rights and duties with respect to one another, and political rights against the state as a whole” [13,25] (p. 262). I will advance three different substantive principles that, if adopted as part of the rule of law, would demand enforcement of animal welfare legislation: rights, dignity, and vulnerability.

### 4.1. Rights

Given that the examples of thick conceptions above demand the law recognise and uphold human rights, it is easy to envisage a thick and expansive conception of the rule of law that also demands recognition of animal rights, or rights of all living beings. There is, after all, a strong and persistent movement in favour of fighting for specific rights of animals, most notably the Steven Wise and his Nonhuman Rights Project (NHRP) [26]. There have even been some advances in that field, with habeas corpus applications finding some success in Colombia and Argentina by extending the human right of liberty to captive animals [27]. Theorists such as Tom Regan [28] and Gary Francione [29] have articulated alternate theses for extending rights to non-human animals. Francione has recently proposed that “any sensible and coherent theory of animal rights should focus on just *one* right for animals—the right not to be treated as the property of humans” [30]. 

Of course, animal welfare legislation is often at complete odds with the rights-based focus of Francione and the NHRP. There is no animal welfare legislation that protects rights of life and liberty—let alone preventing animals from having property status—because animal welfare legislation is predicated on animals having this status; of humans having the capacity to own, use and kill non-human animals. Indeed, it is difficult to categorise animal welfare legislation as containing any rights at all. As Stucki persuasively argues,

“Animals’ current legal protections [in animal welfare legislation] may meet the minimal conceptual criteria for rights, but they do not perform the characteristic normative function of rights. They are, therefore, at best atypically weak and imperfect rights”.

[27] (p. 551)

This means that while it may be possible to include the rights of animals as a substantive conception of the rule of law, such a conception would likely necessitate the repeal of animal welfare legislation. While I do not doubt this as an ideal outcome, it is a far stronger claim than that which I am advancing here, which is simply that the rule of law demands the enforcement of this legislation. The enforcement of legislation that undermines the rule of law might be a more desirable state of affairs than its underenforcement, but it will still undermine the rule of law.

### 4.2. Dignity

Thus, given the logical problem with expanding a rule of law conception to include protection for fundamental animal rights—that entail a far stronger outcome than the claim I wish to make—it is necessary to take an alternate path. One such path is to prove something a little more abstract than the proposition that animals have fundamental rights, viz. that animals have dignity. If it is possible to show that animals have some claim to be treated with dignity, it may be possible to advance a rule of law conception that also seeks to enhance and respect that dignity.

In some ways, it is far more straightforward to prove animals’ claim to dignity than it is to prove that they possess fundamental rights. Unlike fundamental rights—life and liberty, for instance—legislation already recognises animals’ claim to dignity: Switzerland’s constitution and its animal welfare legislation both recognise the dignity of animals [31]. Moreover, as Kempers has argued, despite the conceptual vagueness of the term in the abstract, there is clarity about what such a claim demands in practice: that animals have direct moral relevance, and that legal decision-makers must give their interests due consideration [32]. That means that even if legislators do not go as far as the Swiss by explicitly recognising the dignity of animals, any legislation that appropriately recognises the moral relevance of animals and their interests implicitly recognises their dignity.

Examples of such recognition are multitudinous; what Kempers regards as “traces” of dignity found in the positive law [26] (pp. 183–185). Those traces make it a much more suitable vehicle to advance the interests of animals than more radical projects recognising rights, since it would “not require a transition to an entirely different legal paradigm but rather a shift of emphasis” from irretrievably anthropocentric ideas of animal welfare to more animal-centric ideas of their inherent worth and moral relevance [32] (p. 183). New Zealand, for example, does not recognise the dignity of animals, but does recognise their sentience [33]; interpreting that inclusion as recognising the inherent moral worth of animals and their interests is not such a radical implication. This means that even if there are good philosophical arguments against extending the concept of dignity beyond humans to animals [34,35], given there are equally strong arguments that show it is possible and desirable to do so [36], at the very least, it is not as radical or paradigm-shifting a claim as that of animal rights.

However, even if it possible to show that animals have a claim to dignity, it is the next step—that this justifies an expansive conception of the rule of law—which is the most difficult to make. I take heed of Eisen’s observation that:

“[C]onstitutional theories and institutions justified through appeals to human dignity are not easily retrofitted to meet the demands of justice for animals”.

[37]

This is immediately highlighted by Waldron’s claim that the rule of law can (and should) achieve the “dignity” of individuals by using animals’ lack of dignity as a counterpoint. He argues that practical, procedural aspects of respecting the rule of law amount to respect for the “active intelligence” of the people who are subject to the law and its coercive effects [38]. If the rule of law rests upon respect for the freedom and dignity of each person who is subject to the law, then certain procedural safeguards are integral to any legal system [38]. In explaining his reasoning, he notes:

“Applying a norm to a human individual is not like deciding what to do about a rabid animal or a dilapidated house. It involves paying attention to a point of view and respecting the personality of the entity one is dealing with. As such, it embodies a crucial dignitarian idea—respecting the dignity of those to whom the norms are applied as beings capable of explaining themselves”.

[38] (p. 16)

Waldron would thus exclude non-human actors (or those who lack capacity to explain themselves) from the scope of these requirements. The rabid animal—incapable of explaining itself—is not worthy of respect or dignity under this view.

Yet, as Eisen identifies, some have attempted to “reinterpret constitutional dignity as a value capable of supporting attention to the interests of animals” [38] (p. 573), [39]. Moreover, Eisen argues that although there are many difficulties with extending constitutional dignity to animals, “through iterative processes of public debate and judicial interpretation, animal dignity may come to support clearer substantive demands” [38]. Here, the substantive demands I wish to advance are not significant, but they are clear.

My claim is simple: the dignity of animals means their interests are relevant to how the government decides to enforce legislation that affects those interests. Systemic underenforcement of animal welfare legislation is a signal from government that it undervalues the interests of animals, and means that offending against animals is often undetected and unprosecuted. This maligning of their interests in this way is, using the definition above, an affront against their dignity.

Although Waldron rejects any claim about the dignity of animals, it need not be mutually exclusive with a claim about the dignity of humans in the way he posits. As he himself observes:

“[t]here is an implicit commitment to dignity in the tissues and sinews of law—in the character of its normativity and in its procedures—and we do well not to sell this short by pretending that dignity is a take-it-or-leave-it kind of value”.

[40] (p. 222)

Dignity is not a binary concept, and a claim that underenforcement of animal welfare legislation is an affront to the dignity of animals is not equating animals’ interests with that of their human counterparts. Instead, it is simply holding the state to a standard of conduct and explaining why it has fallen short of that standard. Waldron goes on to explain what this means:

“[a] legal system is a normative order, both explicitly and implicitly. Explicitly it commits itself to certain norms—the rules and standards that it says publicly it will uphold and enforce. Some of these it actually upholds and enforces, but for others, in certain regards, it fails to do so: the law says that the state should pay a pension or should pay compensation to Smith, but Smith does not receive it. The explicit content of the legal system provides us with a pretty straightforward basis for saying on these occasions that the legal system has fallen short of its own standards”.

[40] (p. 221)

Thus, the recognition of the dignity of animals, and including that as a substantive concept within the rule of law means that the systemic failure to the enforce the animal welfare legislation—ignoring and subjugating their interests—an affront to their dignity—undermines the rule of law.

### 4.3. Vulnerability

A similar—but different—substantive value to the recognition and protection of animals’ dignity within the rule of law is to recognise animals as vulnerable, and that a commitment to the rule of law demands a protection of that vulnerability. Ani Satz and Maneesha Deckha have used the language of vulnerability as an alternative entrypoint for animals to become part of the moral community: we should value animals not for their proximity to humans but, instead, because of their vulnerable/precarious lives [41,42]. There is an extensive tradition on considering the vulnerability of human actors, but as Satz notes, unlike humans

“The dependency of nonhuman domestic animals is permanent. Throughout their lives, domestic animals rely on humans to provide them nourishment, shelter, and other care. The permanent dependency of domestic animals is created and controlled by humans, rendering them uniquely vulnerable to exploitation. Domestic nonhuman animals are, for this reason, perhaps the most vulnerable of all sentient beings.”.

[42] (p. 80)

Crucially, however, vulnerability analysis is not contingent on removing the status of animals as property, even if that remains the ultimate goal, avoiding the difficulties identified with the rights analysis described above. Ultimately,

“vulnerability can contribute to mitigating some of the stark effects that the property status entails for the objectification of animals”.

[41] (p. 64)

Deckha uses the example of the Alberta Court of Appeal’s decision in *Reece v. Edmonton* (*City*) to show how a vulnerability paradigm can work in practice. *Reece* focused on whether a non-governmental organisation had standing to bring a private prosecution against the city of Edmonton for its alleged mistreatment of a zoo elephant [43]. Although the majority of the Court held against the NGO, in a powerful dissenting judgment, Chief Justice Fraser uses vulnerability to highlight the importance of vindicating the interests of the elephant that would occur through granting the NGO standing. As Deckha notes, Chief Justice Fraser

“places the issue of proper animal treatment at the same level with the interests of marginalized human groups by writing: “Just as one measure of society is how it protects disadvantaged groups, so too another valid measure is how it chooses to treat the vulnerable animals that citizens own and control”.

[41] (p. 68)

In this way,

“vulnerability provides a language that can advance animals’ interests in a non-instrumental fashion without suppressing animals’ own array of differences or insisting on their similarities to humans”.

[41] (p. 64)

*Reece* shows there is significant potential for the viability of vulnerability to act as a substantive value that forms part of the rule of law. If we recognise that animals are vulnerable members of the moral community, then the state has a special obligation to ensure their interests are protected. The easiest way—under the current law—to protect those interests is to ensure that the state enforces the law; ensuring that when animal interests are contravened, there are consequences.

My claim in Section 3 above was that the legal system (and thus, the state) breaches the rule of law when it fails to enforce the law as written, regardless of that statute. My claim in this section is that the state has a special obligation to enforce animal welfare law in particular. If we include substantive values of advancing and or protecting the dignity or vulnerability of animals in a ‘thicker’ conception of the rule of law, then in order to uphold those values and the rule of law, the state must enforce the legislation that protects those interests.

## 5. Practical Consequences

So far, I have established that the state has a general obligation to enforce the law, and, if we extend the concepts of dignity and vulnerability to animals, a particular obligation to enforce legislation that protects their interests. The systemic underenforcement of animal welfare legislation is a failure to meet that obligation, and in so doing, the state undermines the rule of law. In this section, I will consider the practical implications of these propositions, and specifically, whether the existence of prosecutorial discretion and private prosecutions undermine the validity of my overall argument.

### 5.1. Prosecutorial Discretion

Scarcity of resources means the state cannot—and is not obliged to—enforce the law in every situation. Discretion is necessary to serve the best interests of justice and to ensure the efficient allocation of those scarce resources. Does the importance and necessity of discretion pose a problem for the proposition that the state is under a general obligation to enforce the law?

It is clear that “day-to-day operation of law enforcement and the criminal justice system” depends on the exercise of discretion [44]. As an omnipresent part of that system, discretion is thus integral to facilitating the role of state actors in enforcing the law [45]. This is why “police decisions regarding which crimes to investigate, which persons to pursue, and which persons to arrest have come under judicial review only in the most egregious of circumstances” [46]. The Supreme Court of Canada, for instance, in *R v. Power* held that: “[i]t is manifest that, as a matter of principle and policy, courts should not interfere with prosecutorial discretion” [47] (at 621–623). The latitude that state enforcement agencies have is significant. In New Zealand, for example, 40 per cent of police apprehensions are dealt with by alternative processes which do not lead to prosecution [48]. As mentioned earlier in the paper, in many situations these alternative processes—ranging from educational and supportive responses to formal warnings by police—are appropriate and may achieve broader social justice outcomes [8,9]. 

However, the heads of both the English and New Zealand judiciaries have voiced concern about the inconsistency of law enforcement agencies when exercising this discretion. In England, Lord Chief Justice Judge, expressed concern that out-of-court resolution of criminal offending risked eroding public confidence in the “open and transparent administration of justice” and creating a parallel justice system where “which police officers act as prosecutor, jury, and judge” [49]. In New Zealand, Chief Justice Elias, noting the development of programmes designed to reduce the amount of prosecutions, noted the risks of inconsistency and discrimination, concluding that: “[w]e should be very cautious about going down a path which relies heavily on law enforcement agencies to decide the laws they enforce and the manner of enforcement” [48] (p. 130).

In Canada, even the *Power* court understood that the latitude it gave to prosecutors was at the cost of transparency:

“the confidential nature of the charging process serves important institutional functions, including rehabilitative goals and the goal of increasing general deterrence. The latter is met only by preventing the public from knowing which crimes will be given emphasis in enforcement”.

[47] (p. 626)

That lack of transparency is ultimately a cause for concern:

“[p]rosecutors’ discretionary powers affect the most sensitive interests of the general public. Prosecutors’ decisions are all but unreviewable. As these decisions are made in sites of “low visibility,” maintaining accountability requires a consistent-and potentially uncomfortable-measure of sunlight”.

[50]

The use of discretion when enforcing the law is thus not an unqualified good, and a central concern is a lack of transparency and accountability. Beyond that general concern, however, is it legitimate for state actors not to enforce the law given resource constraints? The American Bar Association cites “the fair and efficient distribution of limited prosecutorial resources” as a factor a prosecutor may properly consider in exercising discretion to initiate, decline, or dismiss a criminal charge [51], and the House of Lords has held that resource constraints were a relevant consideration in police enforcement decision-making [52]. Such constraints are of acute concern with regards to animal welfare offending, because unlike other offending, enforcement comes with a unique expense: the costs of care for animals seized from offenders, which create “a logistical nightmare for the state” [53]. Those costs are so significant that they have led to bespoke “costs of care” legislation requiring defendants to pay for the costs of seized animals whilst they await trial [54]. Such legislation is hardly commonplace, however, meaning the burden often falls on the state or humane society, placing further pressure on an enforcement agency’s decision and their discretion not to prosecute.

Consideration of resource constraints in individual cases, however, is starkly different from the resource considerations preventing enforcement in *all* cases. We know that such blanket policies raise the rule of law concerns identified in Section 3 of this paper. As Joseph Raz notes: “[t]he prosecution should not be allowed, for example, to decide not to prosecute for commission of certain crimes, or for crimes committed by certain classes of offenders. The police should not be allowed to allocate its resources so as to avoid all effort to prevent and detect certain crimes or prosecute certain classes of criminal” [55]. While discretion is a necessity, there is a point when its selective or over-use becomes an abuse of power.

While the determination of that tipping point is a difficult task, the tension is reflective of the general power of the state and its reciprocal duty it has not to abuse that power. As Amirthalingam observes:

“[t]he special protection afforded to prosecutorial discretion is for a very specific and limited purpose, namely to prevent political interference in prosecutorial decisions relating to the initiation, continuance or disposition of criminal prosecutions. It goes hand in hand with the role of the prosecutor as minister of justice and guardian of the public interest. At its core, it is meant to protect the citizen from the Government, not to enable the Government to use it to enhance the reach of the criminal law or to avoid accountability for its actions”.

[56]

State enforcement authorities can use discretion as a shield when resource constraints prevent them from enforcing the law in particular instances. They cannot use discretion as a sword to abdicate from its responsibility to enforce law completely. What, then, of the situation where an enforcement officer or prosecutor is institutionally prevented from detecting, charging or prosecuting an offence? This is, I argue, what occurs with regards to animal welfare legislation. The divestment of enforcement and prosecution of such legislation to non-government organisations uses discretion as an institutional sword: the state avoids responsibility for enforcing breaches by delegating that responsibility to those NGOs. Privately funded, often through charitable donations, these NGOs often do not have the capacity to sufficiently detect, let alone prosecute, offending [5,6]. The state divestment to NGOs who are not effective alternatives to the state leads to the systemic abdication of enforcement of animal legislation [5,6].

This differentiates the state underenforcement of animal welfare legislation from other criminal offences that are also rarely enforced. For instance, ‘jaywalking’—generally defined as a pedestrian walking “onto a roadway into the path of any vehicle that is so close that it is impracticable for the driver of the vehicle to yield the right of way” [57]—is an offence in name only. Although there are some exceptions, [58] police will seldom enforce the law and issue jaywalkers with a fine for the violating the rules of the road. The use of discretion by police not to enforce a minor offence such as jaywalking is different from police and state enforcement agencies being effectively institutionally prevented from enforcing the law. Even if police have the theoretical capacity to enforce animal welfare legislation, in practice, they do not, and thus the discretion is institutionally and improperly fettered [59].

Moreover, even if state enforcement agencies did have the sole responsibility to enforce animal welfare legislation but discharged that responsibility as seldom as they did with jaywalking, we would rightfully view this as an abdication of their responsibility and an abuse of their discretion. The prevalence of jaywalking, if anything, helps prove the argument I advanced in Section 3 of this paper: the lack of publicity that jaywalking is an offence (given the lack of enforcement), and the lack of congruence between the law as declared and as enforced has deleterious consequences. If the law is rarely, if ever, enforced, a citizen is left without any signal about the consequences of crossing the road illegally, and cannot order their lives accordingly. Citizens jaywalk because there is no social norm against doing so, and there is no social norm because the law is not enforced.

This may seem a flippant example, and I do not advocate for more stringent enforcing of this law. Indeed, it signals the problem of having such a law in the first place: removing jaywalking from the rules of the road is a more preferable situation than retaining it, but leaving it unenforced and causing the rule of law issues identified above. Animal welfare legislation is not in the same category: as explained in Section 4, at the very least, the dignity and vulnerability of animals demands that the law exists, and that it is appropriately enforced.

While discretion is an integral part of any criminal justice system, where that discretion is not freely exercised due to under-resourcing or divestment, it has the potential to erode public trust about the transparency and responsiveness of that system. My argument advanced in Section 3 and Section 4 above does not demand universal enforcement of the law, but nor does the existence of discretion mean that the state can abdicate from its enforcement responsibilities.

### 5.2. Private Prosecutions

The very existence of private prosecutions—viz. prosecutions brought by private citizens rather than the state—assumes that the state does not enforce the law in all situations; if we accept private prosecutions are legitimate, then we must accept that the state does not uniformly and universally enforce the law in every instance where it detects a breach. Does the existence of private prosecutions mean that the State should not bear the ultimate responsibility to enforce the law, legitimizing the abdication of responsibility referred to above?

The question is particularly salient with regards to the enforcement of animal welfare legislation, since private prosecutions have long been a model mooted to close the enforcement gap. This was the solution advanced by Cass Sunstein, who in 2003 was perhaps the first theorist to use the term “enforcement gap” in the animal welfare context. [60]. Sunstein argues that under-enforcement of animal cruelty statutes occurs because the state has the responsibility to enforce them but executes this role poorly, and that curing the inability of private citizens to enforce such legislation would close the enforcement gap [60]. Penny Conlon Ellison advances the same proposition, arguing that civil enforcement mechanisms (allowing private citizens to bring suit against those perpetrating animal cruelty) would amount to “cost-effective, non-controversial means of ensuring more vigorous enforcement of such laws” [61].

This issue also arose in the case of *Reece*, discussed above, since it focused on a non-governmental organisation attempting to bring a private prosecution to enforce animal welfare legislation. Deckha argues that “in making this connection, the dissent elevates the relatively low-level status of anticruelty statutes by imprinting the subject with rule of law importance” [62] (p. 798), and the decision was “tethered to the Appellants’ ability to have the law enforced” [62]. This accords Sunstein’s observation that:

“[m]any of those who ridicule the idea of animal rights typically believe in anticruelty laws, and they should strongly support efforts to ensure that those laws are actually enforced”.

[58] (p. 392)

Neither Sunstein, Ellison nor Deckha link the rule of law with the *state* enforcing the law, just that the law ought to be enforced. 

In this paper, I have departed from this position since I have advanced the idea that outsourcing the enforcement of animal welfare to private entities is actually a *cause* of the enforcement gap, and that it is only the state which has the constitutional responsibility and capacity to close that gap. To show why I insist that state enforcement is necessary, we need only look to a jurisdiction who has adopted a model of private enforcement to show its flaws. In New Zealand the state effectively cedes jurisdiction to the Society for the Protection of Animals (SPCA) for prosecuting animal welfare offending involving companion animals. [5,6] These prosecutions take the form of private prosecutions.

Does the fact that private prosecutions are used routinely in New Zealand to enforce animal welfare legislation indicate that the state need not have the sole responsibility to enforce the law? The New Zealand experience indicates otherwise. The SPCA’s position as prosecutor of first resort in New Zealand is anomalous, and indeed, is often cited as an example of the few areas where private prosecutions continue to flourish [63,64]. More generally, the right to bring a private prosecution is one of “anomalous historical survival” [65], and “seems out of place in a system that possesses a fully functioning, adequately equipped, non-corrupt police force, and an independent prosecuting body” [66].

Little and Sheffield provide a précis of that anomalous history [67]. The industrialisation of the nineteenth century led to increasing lawlessness and a desire by newly propertied classes to have an “economical means, given the expensive system of private prosecutions, to have the law *enforced*. It was in this context that private prosecution societies were organized to defray the high cost of prosecution and to gain access to the criminal justice system of the period” [67]. However, after 1845, such prosecution societies began to wane, and once the State took responsibility of the enforcement of the law—most notably with the creation of Department of Public Prosecutions in 1879—such societies ceased to have necessity and relevance [68].

In 1822, England enacted the world’s first national animal cruelty legislation, colloquially known by its sponsor: *Martin’s Act* [69]. Two years later, the first SPCA was established. As Kean notes,

“The Society did not come into being to campaign for new legislation as such, but rather to ensure that the law which had been passed would be implemented”.

[69] (p. 35)

Without the creation of such a society, the law would not have been enforced. Given the history of prosecution societies detailed above, this should come as no great surprise: such societies were not unique to the enforcement of animal cruelty legislation. This model was exported to and imposed in England’s colonies, including New Zealand.

Given the timing of the first instance of animal cruelty legislation, it is only natural that a prosecution society was necessary for its enforcement: it was enacted during the time of peak popularity of prosecution societies. However, what is surprising is that these societies—and this enforcement model—persisted long after it fell out of favour in the rest of the criminal law. Debate now focuses on the whether the residual right to bring a private prosecution should survive a criminal justice system [70], never on whether the tool is legitimate for use in an entire area of the law.

The New Zealand model becomes more anomalous by the day. Most recently, the SPCA has divested itself of its role as prosecutor in the United Kingdom [71]. As Coulter notes, Canada is a patchwork of different approaches, but there may be small signs of the beginning of a trend toward state enforcement, rather than a private enforcement [72]. Most notably, the decision of the Ontario Superior Court of Justice in *Bogaerts v. Attorney General of Ontario* [73] held that investigatory and enforcement powers of that province’s SPCA were unconstitutional. Although that case was overturned on appeal [74], the SPCA nevertheless voluntarily ceded that enforcement responsibility to the province [75]. Similarly, the Edmonton Humane Society withdrew from enforcement operations in 2019, forcing the municipal police to bridge the shortfall [59,72]. The United States is similar, with humane societies sometimes assisting in animal cruelty investigations but never having sole responsibility for doing so [72].

Moreover, private enforcement of animal welfare legislation—including private prosecutions—is a poor facsimile of the state apparatus. Similar to the discussion about discretion above, it is not so much the existence of private prosecutions that represents a concern for my argument, but instead their overuse due to systemic under-resourcing. As Coulter and Campbell note,

“SPCAs and humane societies are motivated by a commitment to protecting animals but have smaller workforces and less resources, funding, and enforcement tools than public policing agencies”.

[7] (p. 516)

A system that relies upon them is thus, almost necessarily, doing a poorer job of enforcement compared to a pure state-enforcement model. Any divestment by the state to private organisations is thus a failure to meet the rule of law obligations identified in Section 3 and Section 4. 

The existence of discretion at all stages of the criminal justice process and private prosecutions are linked in that they indicate that the state is not under a universal, mandatory obligation to enforce the law. This does not, however, mean that from a systemic perspective, enforcement is optional. Applied to animal welfare enforcement, the divestment of the enforcement and, sometimes, prosecutorial function from state to non-state actors, leads to systemic fettering of the discretion and reliance on an anachronistic right to private prosecution. Both are symptoms of a system that runs against the constitutional grain.

## 6. Conclusions

Animal welfare legislation is underenforced. The state consistently abdicates from the enforcement of such legislation in a way rarely found elsewhere in the criminal law. The effect of delegating the enforcement—and sometimes, prosecution—responsibility to non-governmental organisations with limited resources is that contraventions of animal welfare legislation are regularly undetected and rarely prosecuted. How do we assess this enforcement gap?

In this paper, I have proposed that this enforcement gap is of constitutional concern; its persistence is contrary to the rule of law. I have done so in two parts. First, I have proposed that the rule of law imposes a general obligation on the state to enforce the law: the requirements of publicity and congruence work in tandem, such that without enforcement, citizens cannot trust the law as it is written, and cannot plan their lives accordingly. Second, I have proposed that the rule of law imposes a particular obligation to enforce animal welfare legislation. Such an obligation is not contingent on proving that animals have fundamental rights, but instead showing that protecting the dignity and/or vulnerability of animals is a requirement of the rule of law. Respecting animals’ dignity and vulnerability does not mean they are of the same moral standing as humans; simply that they have some moral significance, and that legal decision-makers must take their interests into account. If we can accept that the rule of law has the capacity to include these substantive values, the underenforcement of animal welfare legislation—in subjugating their interests—denies their dignity, fails to protect their vulnerability, and is thus contrary to the rule of law.

Finally, I have addressed the concern with the logical consequence of these propositions. Is the state under an obligation to enforce animal welfare legislation in every circumstance? The existence of discretion in enforcement and private prosecutions would suggest otherwise. In response, I have argued that: first, discretion is necessary but the state cannot use it as a sword to abdicate all enforcement responsibility; and second, private prosecutions are an anachronistic relic—an exception that proves the rule of the state as the enforcer of first resort.

The propositions I have advanced show that animal welfare underenforcement is not simply a problem that should concern those with animal interests in mind, but those also who have an interest in maintaining the rule of law and ensuring our legal system in good working order.

## Data Availability

The data presented in this study are available on request from the corresponding author.
